# LncRNA H19 promotes the committed differentiation of stem cells from apical papilla via miR-141/SPAG9 pathway

**DOI:** 10.1038/s41419-019-1337-3

**Published:** 2019-02-12

**Authors:** Zehan Li, Ming Yan, Yan Yu, Yanqiu Wang, Gang Lei, Yin Pan, Na Li, Romila Gobin, Jinhua Yu

**Affiliations:** 1 Key Laboratory of Oral Diseases of Jiangsu Province and Stomatological Institute of Nanjing Medical University, 140 Hanzhong Road, Nanjing, Jiangsu 210029 China; 2 Endodontic Department, School of Stomatology, Nanjing Medical University, 136 Hanzhong Road, Nanjing, Jiangsu 210029 China

## Abstract

Long noncoding RNAs (lncRNAs) exert significant roles at transcriptional and post-transcriptional levels. Stem cells from apical papilla (SCAPs) differentiate into dentin/bone-like tissues under certain conditions. So far, whether lncRNA-H19 can affect the proliferative behaviors and osteo/odontogenesis of SCAPs, as well as its specific mechanism remain to be elucidated. Here, SCAPs were isolated and transfected with the lentiviruses or packaging vectors. Our results showed that lncRNA-H19 had no significant effect on the proliferative behaviors of SCAPs, as presented by CCK-8 assay, EdU assay and flow cytometry (FCM). Furthermore, alkaline phosphatase (ALP) activity, alizarin red staining, Western blot assay (WB), quantitative real-time polymerase chain reaction (qRT-PCR) and in vivo bone formation assay were conducted to verify the biological influences of H19 on SCAPs. Overexpression of H19 led to the enhanced osteo/odontogenesis of SCAPs, whereas knockdown of H19 inhibited these effects. Mechanistically, H19 competitively bound to miR-141 and prevented SPAG9 from miRNA-mediated degradation, thus significantly elevating phosphorylated levels of p38 and JNK and facilitating the committed differentiation of SCAPs. Taken together, the osteo/odontogenesis of SCAPs was upregulated by overexpression of H19 via miR-141/SPAG9 pathway.

## Introduction

Regeneration of the lost bone is very important in diseases with bone loss, such as tumors, bony defects and fractures. In recent years, mesenchymal stem cells (MSCs)-based cellular therapies present a promising prospect for bone defect treatment^1^. MSCs could differentiate into a variety of adult cell types including osteoblasts. Due to their strong multi-potentiality and regenerative properties, biological functions of MSCs have been well recognized and their researches on bone tissue engineering achieved great process^2,3^. Moreover, they can be isolated from numerous tissues in humans, such as peripheral blood, bone marrow, umbilical cord blood, placenta, and dental tissues^4^. However, in comparison with other sources, MSCs derived from dental tissues exist in the human body during the whole life. In addition, it is generally believed that they are extremely accessible. The isolation of MSCs from dental tissues is convenient during the procedures. Hence, they are considered to be potent candidates for bone tissue engineering^5,6^.

As a major kind of dental stem cells, stem cells from apical papilla (SCAPs) are essential for the developing alveolar bone, tooth root and dental pulp-dentin complex. They are isolated from the soft tissues at the apices of developing permanent teeth^7^. SCAPs exert advantages of self-renewing and multilineage differentiation such as osteogenic, odontogenic, adipogenic, and neurogenic^8^. It has been reported that SCAPs present remarkable tissue regenerative capability in spinal cord injuries^9^. Besides, a relative study conducted using the swine model showed the biological function tooth root produced by SCAPs^10^. Complex molecular mechanisms including signaling pathways and microRNAs underlying SCAPs osteo/odontogenic differentiation have been extensively investigated^11,12^. Our previous studies have demonstrated that many factors including growth factors (e.g., insulin-like growth factor I, IGF-I)^13^, bioactive materials (e.g., mineral trioxide aggregate)^14^, and hormones (e.g., 17beta-estradiol)^15^ can affect the osteo/odontogenic differentiation of SCAPs.

In the past decades, long noncoding RNAs (lncRNAs) have exerted their biological functions in the transcriptional and post-transcriptional regulation of diverse biological processes, such as cellular progression and differentiation^16,17^. Recently, lncRNA expression profiles analyzed by the high throughput technologies characterized a number of osteogenesis-related lncRNAs. For example, lncRNA-TUG1 accelerates osteogenic differentiation in periodontal ligament stem cells^18^. LncRNA-MEG3 stimulates osteogenic differentiation of MSCs as well^19^. LncRNA-ANCR inhibits osteogenesis through physical interaction of EZH2 and direct regulation of Runx2^20^. Recent studies have demonstrated that lncRNAs could serve as competing endogenous RNA (ceRNA) by interacting with the miRNA, thus regulating target gene expression^21,22^.

As we all know, microRNAs (miRNAs) are major players in gene regulation through binding to the 3′-untranslated region (3′UTR) of the target mRNAs, and subsequently cause mRNA degradation or translation inhibition^23^. LncRNA serves as a miRNA spong and relieves inhibitory effect of miRNA on target genes. For example, lncRNA-1604 sponges to miR-200c, leading to ZEB overexpression and thus promotes embryonic stem cells differentiation^24^. LncRNA TUG1 regulates the expression of its target *FGF1* by sponging miR-133a^25^. LncRNA-H19 is of great significance in promoting skeletal muscle differentiation as one of the most conserved noncoding transcripts in mammalian development^26^. Despite the previous achievements, the specific mechanism of H19 in influencing osteo/odontogenic differentiation of SCAPs remains unknown. Here, we demonstrated for the first time that H19 promoted the osteo/odontogenic differentiation of SCAPs while miR-141 inhibited. Moreover, H19 sponged miR-141 and released its inhibitory effect on SPAG9. Our results provide references for further analysis of the lncRNA-miRNA-mRNA network during the regeneration of the bone/dentin tissues.

## Materials and methods

### Cell culture

This study got approval of the Ethical Committee of the Stomatological School of Nanjing Medical University. Experimental procedures were conducted in accordance with the Human Care Guidelines of the Ethical Committee of Nanjing Medical University. Impacted third molars were collected from 9 healthy donors aged 17-20 years after the informed consent was obtained in the Oral Surgery Department of Jiangsu Provincial Stomatological Hospital. The apical papilla were carefully isolated from the immature roots, cut and digested in medium containing 3 mg/ml collagenase type I and 4 mg/ml dispase (Sigma, St. Louis, MO, USA) at 37 °C. Thirty minutes later, cells were purified using rabbit anti-STRO-1 antibody (Santa Cruz, Delaware, CA) and sheep anti-rabbit IgG Dynabeads (Dynal Biotech, Oslo, Norway) followed by magnetic activated cell sorting (MACS) instructions. Isolated cells were maintained in alpha minimum essential medium (α-MEM, Gibco, Life Technologies, Grand Island, NY, USA) with 10% fetal bovine serum (FBS, Hyclone, Logan, UT, USA), 100 U/mL penicillin and 100 mg/mL streptomycin at 37 °C in a 5% CO_2_ incubator. Culture medium was replaced every other day. Based on the cell surface makers, SCAPs were identified by flow cytometry (FCM) as previously described^15^. Third-passage cells were harvested for the subsequent experiments.

### Lentivirus infection

Recombinant lentiviruses containing full-length H19 (Gene Bank accession number, NR_002196.1) and scramble control (NC) were obtained from GenePharma Company (Shanghai, China). Recombinant lentiviruses targeting H19 (Lenti-shH19-1 and Lenti-shH19-2) and scramble control (Lenti-shNC) were also obtained from GenePharma Company. SCAPs were transfected by lentiviruses exposure in 1 mL α-MEM supplemented with 10% FBS and 8 μg /mL polybrene (POL) for 10 h. Infected cells were cultured in the conventional medium and the expression level of H19 was detected.

### Plasmid construction and transfection of miRNA mimics/inhibitors

MiRNA plasmids were obtained from Ribobio Company (Guangzhou, China). SCAPs were transfected by using transfection reagent riboFECT^TM^ CP (Ribobio, Guangzhou, China). The mutated binding sites of miR-141 in luciferase reporter vectors containing H19 and SPAG9 were constructed by site-directed mutagenesis. Transient transfection was conducted using Lipofectamine 2000 (Invitrogen, USA).

### Flow cytometry

Transfected cells were collected by using trypsin (Beyotime, Haimen, China) and fixed with pre-cold alcohol at 4 °C overnight in dark. After phosphate buffered saline (PBS) wash, cell cycle phases (G0/G1, S, and G2/M phases) were evaluated using FACScan flow cytometer (BD Biosciences, San Jose, CA). The experiment was repeated three times.

### Cell proliferation assay

Regulatory effects of H19 on proliferative potential were determined by the Cell Counting Kit-8 (CCK-8 kit) (Dojindo, Tokyo, Japan) assay and EdU incorporation assay. Briefly, transfected SCAPs were plated into a 96-well plate with 3 × 10^3^ cells/well. 10 μL CCK-8 reagent was added at different time points (day 0, 1, 3, 5, 7, 9, respectively), and the absorbance at 450 nm was measured 2 h later by a microplate reader. The experiment was repeated in triplicate.

For EdU incorporation assay, transfected SCAPs (5 × 10^3^ cells per well) received 2-h incubation with25 mM 5-ethynyl-20-deoxyuridine (EdU, Ribobio). After fixation in 4% paraformaldehyde (PFA) for 15 min and induction with 0.5% Triton X-100 for 20 min at room temperature, cells were treated with 1 × Apollo reaction cocktail for 30 min. Subsequently, the DNA was stained with Hoechst 33342 for 20 min and visualized using a fluorescence microscope.

### Alkaline phosphatase (ALP) activity and staining

ALP activity was recorded at 405 nm using an ALP activity assay kit (Jiancheng, Nanjing, China) as previously described^14^. Total protein content of each sample was determined with a BCA kit (Beyotime, China). ALP activity relative to the control group was normalized to the total protein content.

According to the protocol of the NBT/BCIP staining kit (Beyotime, China, transfected SCAPs were washed with PBS and fixed in 4% PFA for 30 min. After PBS wash for three times, cells were incubated in alkaline solution for 20 min at 37 °C.

### Alizarin red staining and quantification

As described previously, alizarin red staining was performed to evaluate mineralization^13^. Transfected SCAPs were fixed in ice-cold 70% ethyl alcohol for 30 min and stained with 40 mM/L alizarin red (pH = 4.2, Sigma-Aldrich) for 20 min at room temperature. Alizarin red dissolved in 10 mmol/L sodium phosphate containing 10% cetylpyridinium chloride (CPC, Sigma-Aldrich) for 30 min at 25 °C was used to quantification by the spectrophotometric absorbance at 570 nm. The final calcium concentration was normalized to the total protein content.

### Real-time reverse transcription polymerase chain reaction

We used TRIzol reagent (Invitrogen, New York, NY, USA) to extract cellular RNA. RNA was determined at 230, 260, and 280 nm, respectively. The mRNA was reversely transcribed into cDNA using the PrimeScript RT Master Mix kit (TaKaRa Biotechnology, China). RT-PCR was performed using SYBR Green Master (Roche, Indianapolis, IN, USA) and ABI 7300 real-time PCR system. Primers used in this experiment were listed in Table 1. Human U6 RNA was applied as an internal control. Human *GAPDH* was used as a control for normalizing expressions of osteo/odontoblast-associated genes (*ALP, DSPP, DMP1, RUNX2, OSX,* and *OCN*) calculated by the 2^−ΔΔCt^ method as previously reported^12^.

**Table 1:** Sense and antisense primers for real-time reverse transcription polymerase chain reaction

Genes	Primers	Sequences (5’-3’)
*DMP*1	Forward	CCCTTGGAGAGCAGTGAGTC
	Reverse	CTCCTTTTCCTGTGCTCCTG
*COL-I*	Forward	CCCTTTCTGCTCCTTTCT
	Reverse	TGTTTCCTGTGTCTTCTGG
*RUNX*2	Forward	TCTTAGAACAAATTCTGCCCTTT
	Reverse	TGCTTTGGTCTTGAAATCACA
*DSPP*	Forward	ATATTGAGGGCTGGAATGGGGA
	Reverse	TTTGTGGCTCCAGCATTGTCA
*OSX*	Forward	CCTCCTCAGCTCACCTTCTC
	Reverse	GTTGGGAGCCCAAATAGAAA
*OCN*	Forward	AGCAAAGGTGCAGCCTTTGT
	Reverse	GCGCCTGGGTCTCTTCACT
*H*19	Forward	CTTTCATGTTGTGGGTTCTGG
	Reverse	CGGGTCTGTTTCTTTACTTCC
*SPAG*9	Forward	GGCGGCTCGAGAAAATCCGTTCTA
	Reverse	AATGCGGCCGCAACTCAATCAAC

### Western blot

Cell lysates were harvested by RIPA buffer (Beyotime, China) with Complete Protease Inhibitor Cocktail (Roche, USA). Protein samples were separated by 10% SDS-PAGE and transferred to PVDF membrane (Millipore, USA). The membranes were blocked in 5% BSA for 2 h at room temperature and incubated overnight with primary antibodies [OCN (ab93876, Abcam, UK), OSX (ab22552, Abcam, UK), RUNX2 (ab76956, Abcam, UK), ALP (ab95462, Abcam, UK), DSP (sc-33586, Santa Cruz), ERK (#4695, Cell Signaling Technology), p-ERK (#4370, Cell Signaling Technology), p38 (#8690, Cell Signaling Technology), p-p38 (#4511, Cell Signaling Technology), JNK (#9252, Cell Signaling Technology), p-JNK (#9255, Cell Signaling Technology), SPAG9 (#5519, Cell Signaling Technology) and GAPDH (#2118, Cell Signaling Technology)]. Membranes were washed with Tris-buffer saline containing 0.05% Tween 20 (TBST) for three times and 5 min each. The membranes were incubated with secondary antibodies for 1 h in room temperature, followed by TBST wash for 30 min. Western blot analysis were quantified using ImageJ software (http://rsb.info.nih.gov/ij/).

### Immunofluorescence staining

Transfected SCAPs were seeded onto 10 mm^2^ glass coverslips and cultured for three days. Cells were washed with 0.01 mol/L PBS and fixed with 4% PFA for 30 min at room temperature. After PBS wash, cells were permeabilized with 0.25% Triton-100 for 12 min, and blocked with normal goat serum (DCS/BioGenex, Hamburg, Germany) for 45 min at 37 °C. Incubation of primary antibodies [RUNX2 (ab76956, Abcam, UK), OSX (ab22552, Abcam, UK) and DMP1 (NBP1-45525, Novus)] were conducted for 12 h at 4 °C, followed by secondary antibody labeling with fluorochrome for another 30 min at 37 °C in dark. Coverslips were observed under the inverted fluorescence microscope (Olympus, Japan).

### Dual-luciferase reporter assay

The HEK293T cells were seeded into a 24-well plate with 5 × 10^5^ per well. Cells were co-transfected with luciferase plasmids and miR-141 mimic or negative control. Luciferase activities of Renilla and Firefly were measured 48 h after transfection using Dual Luciferase Reporter Assay System (Promega).

### RNA immunoprecipitation (RIP)

Magna RIPTM RNA-Binding Protein Immunoprecipitation Kit (Millipore, Bedford, MA, USA) was used for RIP assay. Briefly, lysed cells were incubated with RIP buffer containing magnetic beads conjugated with antibodies against JIP-4 (#5519, Cell Signaling Technology) and rabbit IgG control to precipitate the potential substances in the RISC complex. RNA purification was performed using RNase-free DNase I and proteinase K (Thermo Fisher Scientific, Waltham, MA, USA). The mRNA level of miR-141 in extract was detected by qRT-PCR.

### Bone formation assay and micro-CT analysis

SCAPs with H19 overexpression and controls induced under osteogenic medium for 1 w were harvested for the in vivo study. SCAPs cells incubated with Bio-Oss Collagen (Geistlich, Germany) scaffolds for 1 h at 37 °C were implanted into the dorsal sides of BALB/c homozygous nude mice (5 weeks old, five mice in each group). Eight weeks later, implants were harvested and fixed in 4% PFA. Animal procedures got approval by the Animal Care and Use Committee of Nanjing Medical University. Micro-CT analysis was performed using a highresolution Inveon Micro-CT (Siemens, Munich, Germany) set as 80 kV of X-ray source, 500 μA of a node current and 500 ms of the 360 rotational steps per time. Micro-CT image analysis software (Inveon Research Workplace) was utilized for reconstructing image slices. The ratio of new bone volume to existing tissue volume (BV/TV) was calculated.

### Histological and histomorphometric analyses

Tissues were decalcified in 10% ethylene diamine tetraacetic acid (pH 7.4) for 4 w, dehydrated and paraffin embedded. Tissues were sectioned and stained with hematoxylin and eosin (H&E) or Masson’s trichrome. Ten randomly selected fields in each section were captured under the microscope. Immunohistochemical staining for OCN was performed. Decalcified sections were blocked with goat serum, incubated with primary antibodies against OCN (1:300 dilution) at 4 °C overnight, and analyzed with the ABC detection kit (Maixin Biotech). Immunohistochemical staining was captured under the microscope.

### Statistical processing

Data analyzed with were GraphPad Prism software expressed as means ± SD from three independent experiments. Statistical Package for Social Sciences (SPSS) software (version 16.0) was utilized for statistical analyses. Differences between two groups were compared using the Student’s *t* test. *P* < 0.05 indicated statistically significant.

## Results

### LncRNA-H19 expression increases with the prolongation of osteogenic differentiation of SCAPs

**Fig. 1:**
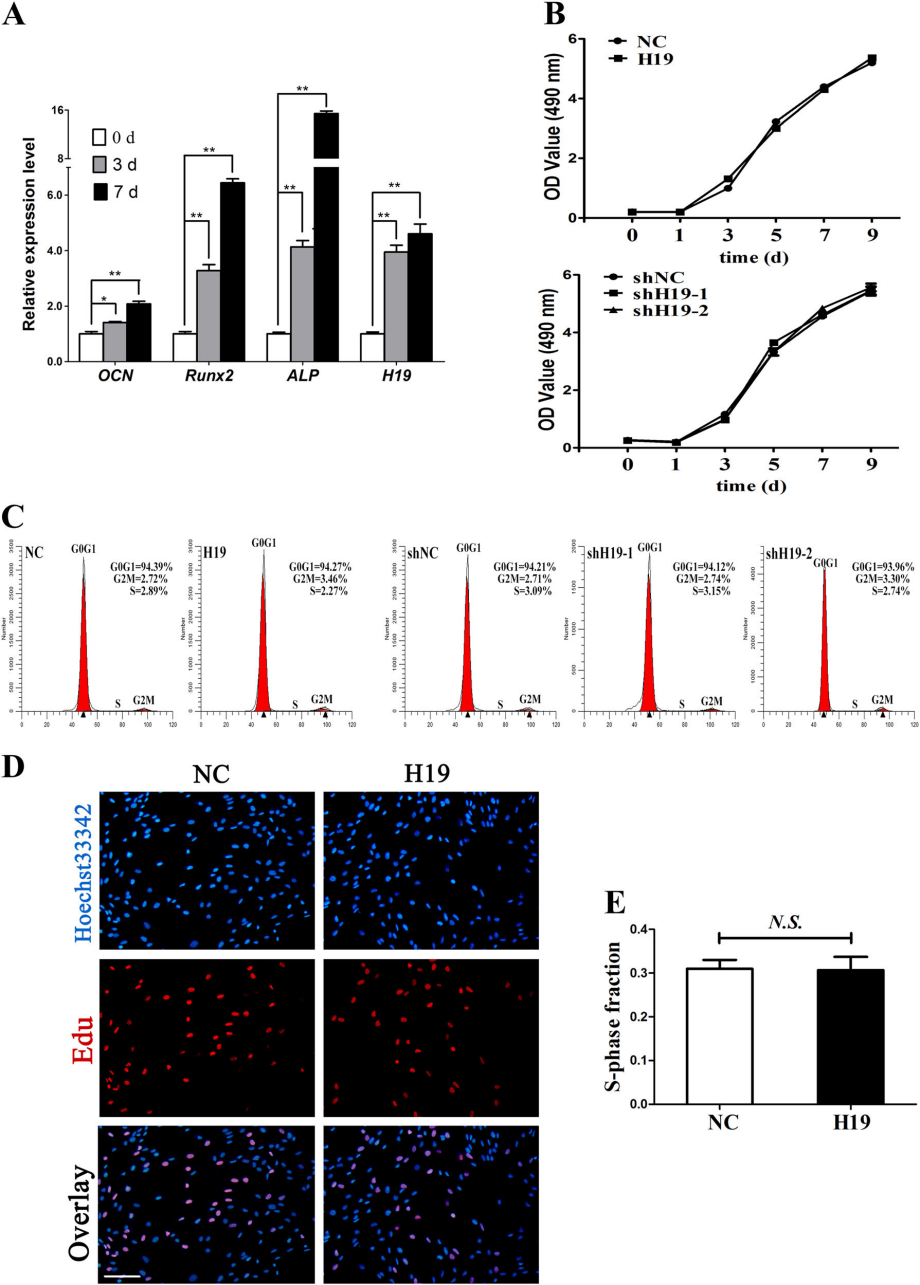
LncRNA-H19 expression during osteoblast differentiation of stem cells from apical papilla (SCAPs) and effects of lncRNA-H19 on proliferation of SCAPs. **a** Relative expression of *H19* and osteoblastic markers of *OCN*, *Runx2*, and *ALP* were determined by qRT-PCR analysis during osteoblast differentiation of SCAPs at day 0, 3 and 7. The relative expression leves at indicated time points were normalized to day 0. GAPDH was used as an internal control. Results were presented as the mean ± SD (*P < 0.05, **P < 0.01). **b** CCK-8 assay showed no significant difference in cell proliferation when H19 was overexpression or knockdown from day 0 to day 9. **c**, **d** Flow cytometry and EdU assay demonstrated that H19 had no significant difference on cell proliferation of SCAPs at day 7 (Scale Bar = 200 μm; N.S., *P* > 0.05)

To identify the underlying effect of lncRNA-H19 on the osteogenic differentiation of SCAPs, RNA samples were collected for detecting expression changes of H19. RT-PCR results revealed that *H19* expression at day 3 and 7 of osteogenic differentiation of SCAPs was gradually upregulated (*P* < 0.05, Fig. 1a). We also examined the mRNA expressions of *ALP* and *RUNX*2 (the early-stage osteogenic markers), which displayed a significant increase in the osteogenic differentiation of SCAPs. Meanwhile, *OCN* (the late-stage marker) was also significantly upregulated with time (*P* < 0.05 or *P* < 0.01, Fig. 1a).

### LncRNA-H19 could not affect SCAPs proliferation

To elucidate the function of lncRNA-H19 in SCAPs proliferation, lentivirus was transfected to alter H19 expression in SCAPs. Stably transfected cells were assigned into NC, H19, shNC, shH19-1, and shH19-2 groups. Transfection efficacy of H19 was confirmed by quantitative RT-PCR analysis (*P* < 0.01, Supplemental Figs. 1A and 1B) CCK-8 assay did not show significant difference in proliferative rate between H19 group and NC group or among shNC, shH19-1 and shH19-2 groups at day 9 (*P* > 0.05, Fig. 1b). FCM analysis did not reveal distinct difference in the proliferative index (PI = G2M ± S) between NC group (5.61%) and H19 group (5.73%, *P* > 0.05, Fig. 1c). Likewise, no distinct difference was found in the proliferative index among shNC group (5.8%), shH19-1 group (5.89%) and shH19-2 group (6.04%, *P* > 0.05, Fig. 1c). Moreover, EdU retention assay showed no obvious difference between NC group and H19 group as well (*P* > 0.05, Fig. 1e). Taken together, the data accumulated here elucidated that lncRNA-H19 could not affect SCAPs proliferation.

### LncRNA-H19 promotes the osteo/odontogenesis of SCAPs

**Fig. 2:**
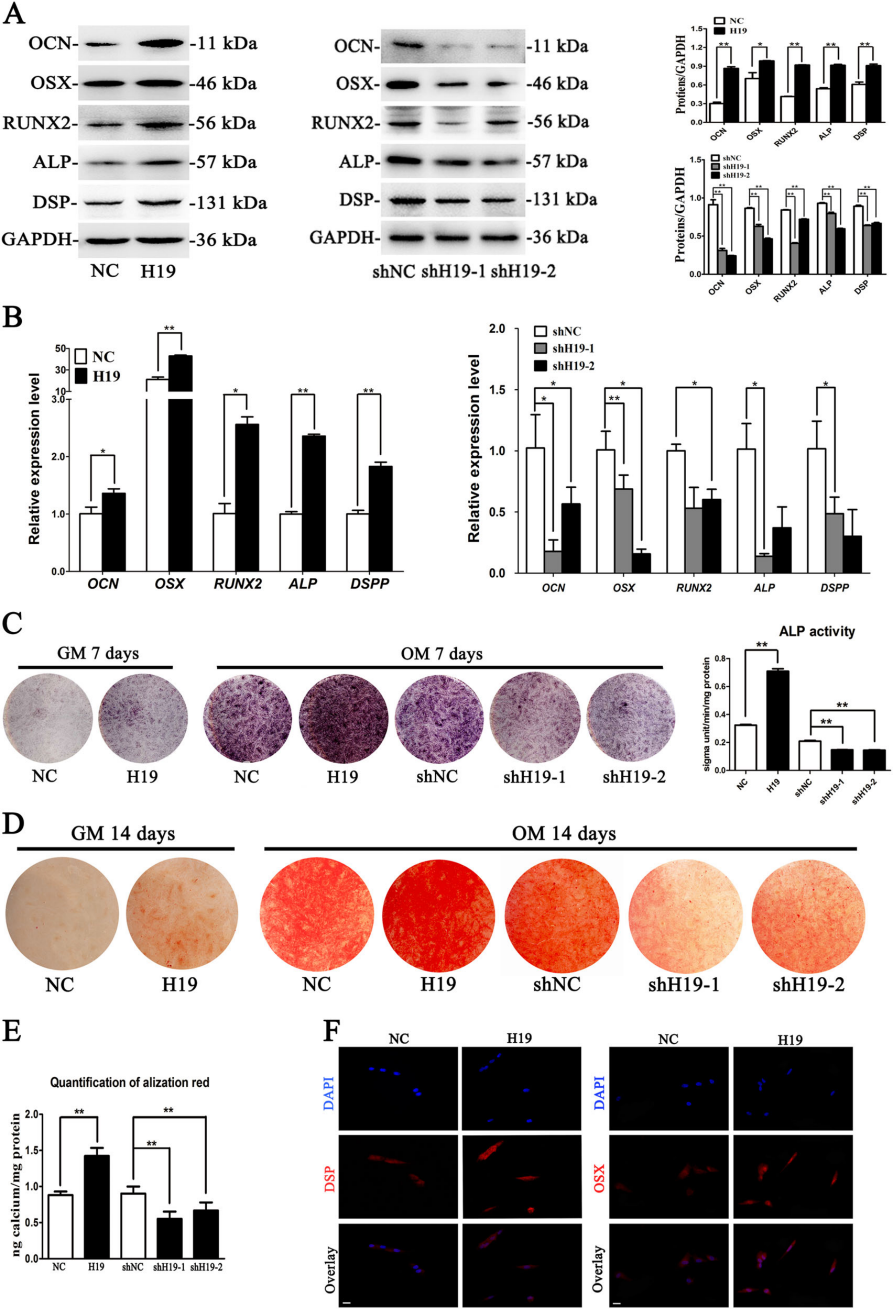
LncRNA-H19 promotes osteo/odontogenesis of SCAPs. **a** Western blot results revealed that the protein levels of OCN, OSX, RUNX2, ALP and DSP significantly increased in H19 group while decreased in shH19-1 and shH19-2 groups after osteoblast induction for 7 days. GAPDH served as an internal control. Histograms showed the quantification of band intensities (*P < 0.05, **P < 0.01). **b** Relative mRNA expressions of *OCN*, *OSX*, *RUNX*2, *ALP* and *DSPP* measured by qRT-PCR after OB induction for 7 days. *GAPDH* was used for normalization. Results were presented as the mean ± SD (*P < 0.05, **P < 0.01). **c** Images of ALP staining in the NC, H19, shNC, shH19-1, and shH19-2 groups. SCAPs were cultured in growth medium (GM) or osteogenic medium (OM) for 7 days. Histograms show the activity of ALP were increased by H19 overexpression and decreased by H19 knockdown after OB induction for 7 days (**P < 0.01). **d** After cell culture in GM or OM for 14 days, alizarin red staining showed that H19 group generated more calcified nodules than control group. shNC group generated more calcified nodules than shH19-1 and shH19-2 groups. **e** Histograms showed quantification of Alizarin red staining by spectrophotometry. Values were expressed as means ± SD, **P < 0.01. **f** Immunofluorescence assay revealed that the expressions of DSP and OSX in Lenti-H19 treated SCAPs were significantly up-regulated (Scale Bar = 50 μm)

Transfected SCAPs were induced in osteoblast differentiation medium, and Western blot results demonstrated that protein expressions of OCN, OSX, RUNX2, ALP, and DSP in Lenti-H19 infected SCAPs were markedly higher than those in control group at day 7 (*P* < 0.05 or *P* < 0.01, Fig. 2a). Meanwhile, the mRNA levels of *OCN*, *OSX*, *RUNX*2, *ALP*, and *DSPP* also increased by H19 overexpression, whereas H19 knockdown obtained the opposite effects (*P* < 0.05 or *P* < 0.01, Fig. 2a, b). ALP activity was obviously upregulated by H19 overexpression at day 7 and H19 knockdown resulted in decreased ALP activity (Fig. 2c). After 14 days of osteogenic induction, ALP activity and matrix mineralization were markedly enhanced (Fig. 2c, d). ALP activity and matrix mineralization were even enhanced in H19 group without osteogenic supplements as compared with controls (Fig. 2c, d). CPC results also revealed that the calcium concentration in H19 overexpressing cells was much higher than those cells with H19 knockdown at day 14 (*P* < 0.01, Fig. 2e). In addition, immunofluorescence staining showed upregulated protein levels of OSX and DSP in Lenti-H19 infected SCAPs as compared with NC group at day 7 (Fig. 2f). These results indicated that H19 promotes the osteo/odontogenic differentiation of SCAPs.

**Fig. 3:**
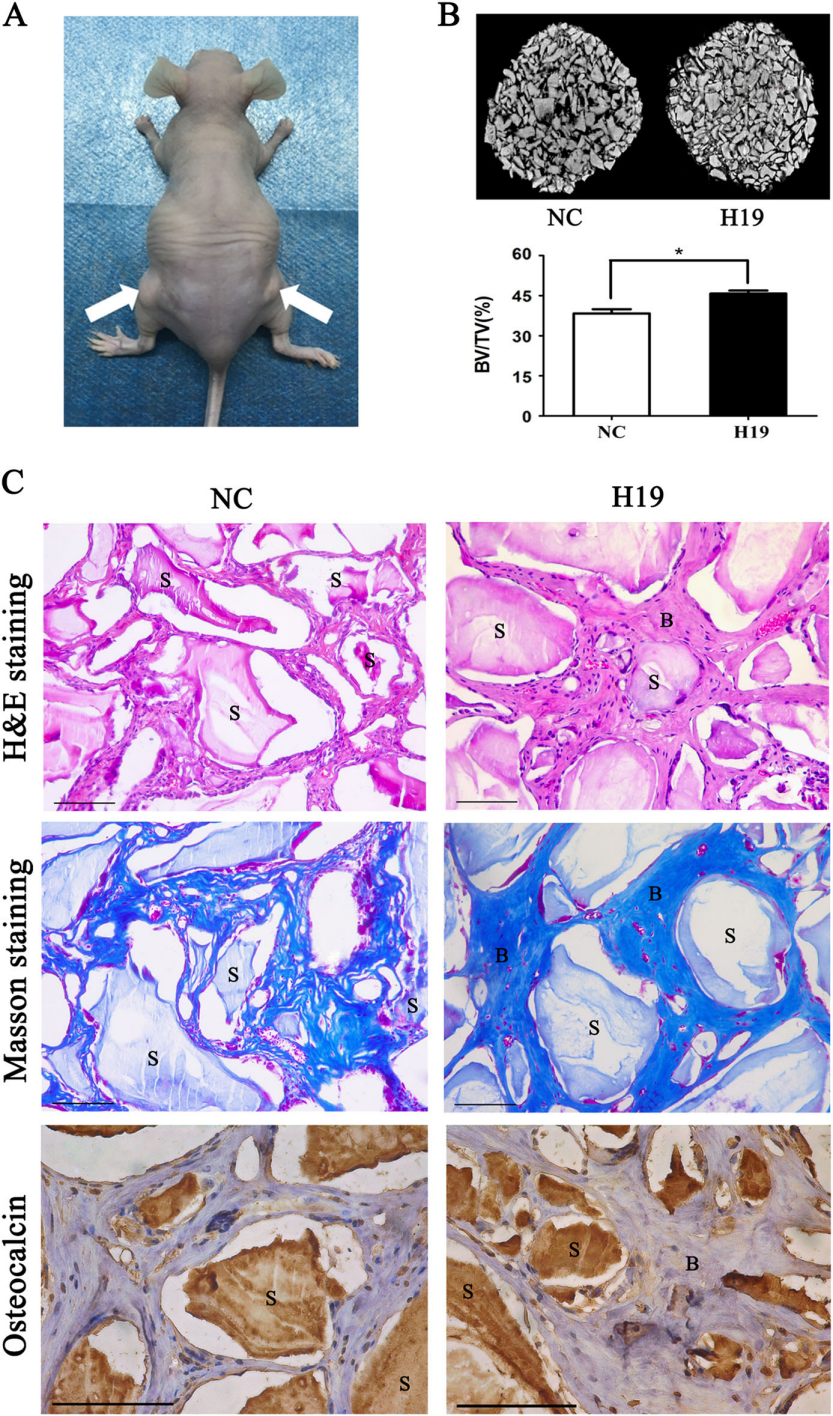
H19 enhanced the osteo/dentinogenesis of SCAPs in vivo. **a** SCAPs in NC and H19 group were transplanted subcutaneously into 5-week-old BALB/c homozygous nude mice for 8 weeks. **b** Upper: reconstructed three-dimensional micro-CT images of the tissue-engineered bone constructs from NC and H19 groups. Lower: percentages of new BV/TV of cultured bone constructs. Data are shown as the mean ± SD (*P < 0.05). **c** H&E staining, Masson staining and immunohistochemical staining of osteocalcin in NC and H19 groups. B bone/dentin-like tissues, S around the scaffold, BV/TV bone volume to tissue volume, NC negative control. Scale Bar = 100 μm

SCAPs stably expressing H19 and controls were loaded on Bio-Oss Collagen scaffolds, and implanted in the subcutaneous tissues of nude mice (five mice in each group) for 8-week growth (Fig. 3a). BV/TV increased in the H19-overexpressing group compared with the control group (Fig. 3b). Histological examination corroborated the result of BV/TV. Both H&E and Masson staining showed more bone-like structures and collagen deposit in SCAPs of the H19-overexpressing group than the control group (Fig. 3c). Moreover, the abundance of OCN increased in the H19-overexpressing group than control group (Fig. 3c).

### LncRNA-H19 serves as a miRNA sponge for miR-141

**Fig. 4:**
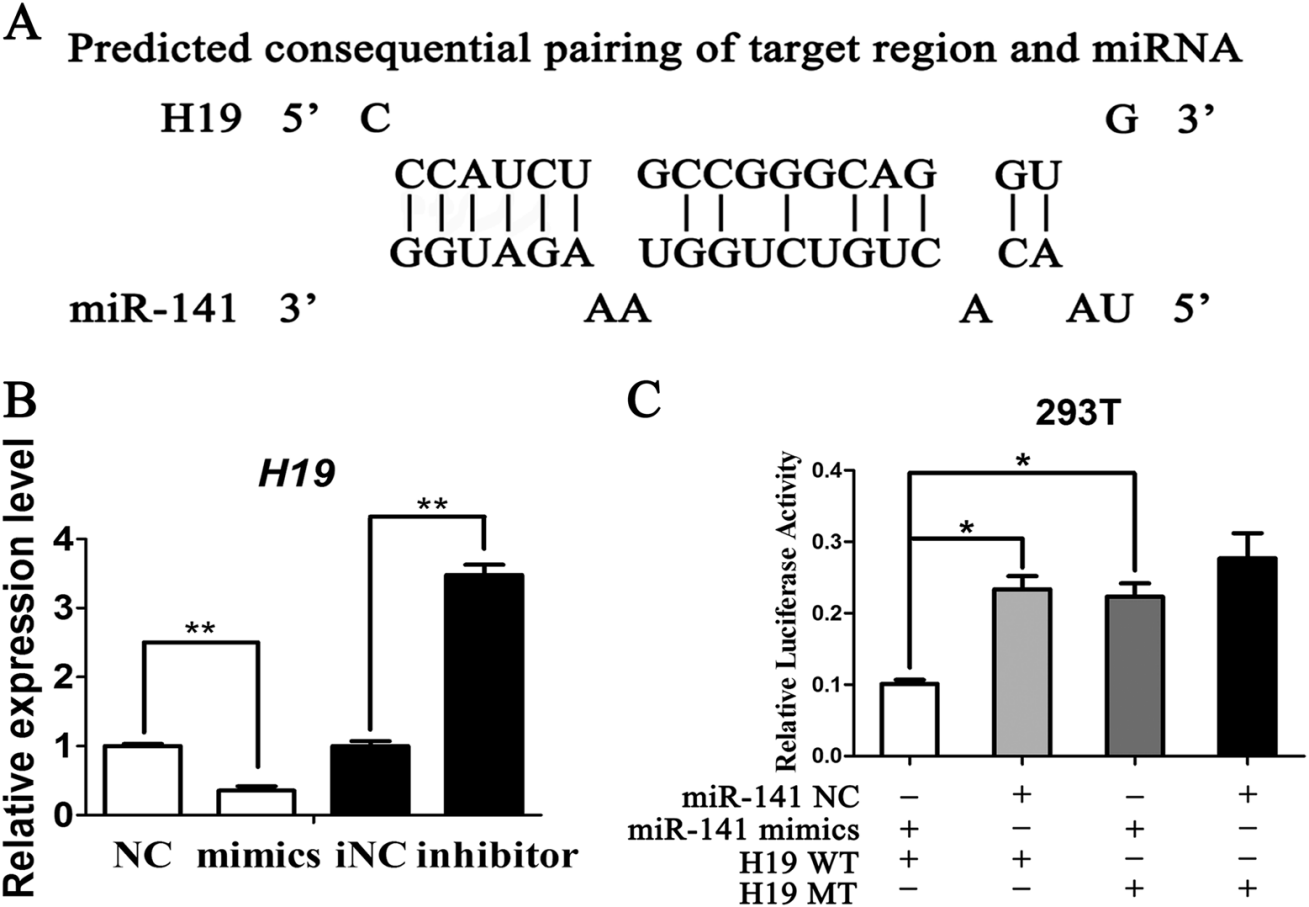
LncRNA-H19 functions as an endogenous sponge of miR-141. **a** The potential binding sites between lncRNA-H19 and miR-141 predicted by biological software. **b** Relative lncRNA-H19 expression level in SCAPs transfected with with miR-141 mimics or miR-141 inhibitor (**P < 0.01). **c** Luciferase reporter assay was used to validate the target in 293T cells. The relative luciferase activities of luciferase reporters containing WT or Mut lncRNA-H19 were assayed 48 h after co-transfection with miR-141 mimics or mimics NC. Relative Renilla luciferase activity was normalized to that of firefly luciferase (*P *<* 0.05, **P < 0.01)

To determine how H19 regulates the osteo/odontogenic differentiation of SCAPs, the candidate miRNAs targeting H19 was searched by miRDB and Target Scan software. MiR-141 was predicted as the potential target of lncRNA-H19 (Fig. 4a). Transfection efficacy of miR-141 was verified by qRT-PCR (Supplemental Fig. 1C). H19 expression was negatively regulated by miR-141 (*P* < 0.01, Fig. 4b). To further determine whether H19 directly regulates miR-141, we cloned the putative miR-141 target binding sequence into a luciferase construct. MiR-141 mimics and H19 wild-type or mutant reporter were transferred into 293T cells. Luciferase activity of H19 wild-type reporter was markedly reduced by miR-141 mimics, and mutation of the putative miR-141 target sites successfully reversed the previous suppressive effect (*P* < 0.05, Fig. 4c). These findings suggested the binding condition between miR-141 and H19.

### MiR-141 inhibits the osteo/odontogenic differentiation of SCAPs

**Fig. 5:**
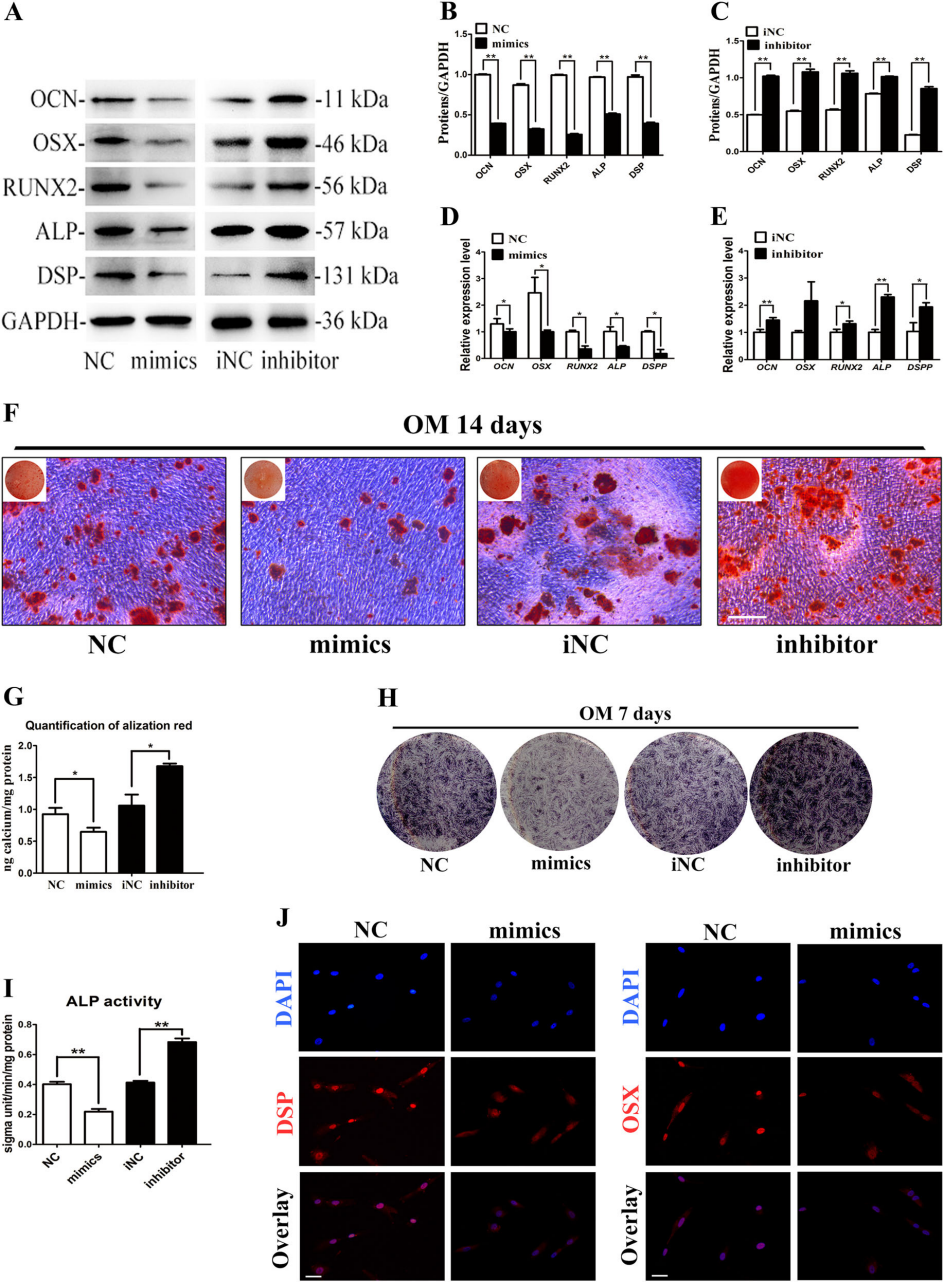
MiR-141 inhibits osteo/odontogenic differentiation of SCAPs. **a**–**c** Western blot results revealed that the protein levels of OCN, OSX, RUNX2, ALP and DSP significantly decreased in miR-141 mimics group while increased in miR-141 inhibitor group (**P < 0.01). **d**, **e** Relative mRNA expressions of *OCN*, *OSX*, *RUNX*2, *ALP*, and *DSPP* were measured by qRT-PCR. *GAPDH* was used for normalization. Results were presented as the mean ± SD (*P < 0.05; **P < 0.01). **f**, **g** After cell culturing in OM for 14 days, alizarin red staining showed that miR-141 mimics group generated more calcified nodules than control group. MiR-141 inhibitor group generated less calcified nodules than iNC group (Scale Bar = 200 μm). Histograms showed quantification of Alizarin red staining by spectrophotometry. Values were expressed as means ± SD, **P < 0.01. **h**, **i** Images of ALP staining in the NC, mimics, iNC and inhibitor groups. SCAPs were cultured in OM for 7 days. Histograms showed the activity of ALP increased by miR-141 overexpression and decreased by miR-141 knockdown (**P < 0.01). **j** Immunofluorescence assay revealed that the expressions of DSP and OSX were significantly up-regulated in miR-141 mimics group compared with NC group (Scale Bar = 50 μm)

To investigate the effect of miR-141 on the osteo/odontogenic differentiation of SCAPs, miR-141 mimics and inhibitor were used to transiently transfect SCAPs. MiR-141 inhibited the osteo/odontogenic differentiation of SCAPs while miR-141 inhibitor increased it at day 7 (*P* < 0.05, Fig. 5a–c). Thereafter, mRNA levels of several osteo/odontogenic marker genes were determined by qRT-PCR. MiR-141 inhibited mRNA levels of majority osteo/odontogenic marker genes (*P* < 0.05 or *P* < 0.01, Fig. 5d, e). In addition, after the osteogenic induction for 14 days, overexpression of miR-141 led to a decreased calcified nodules assessed by alizarin red staining (Fig. 5f), and CPC results further confirmed these results (*P* < 0.05, Fig. 5g). Moreover, ALP analysis also confirmed that miR-141 obviously alleviated the expression of ALP in the committed differentiation of SCAPs (*P* < 0.01, Fig. 5h, i). Immunofluorescence staining revealed that the protein levels of OSX and DSP remarkably decreased in SCAPs transfected with miR-141 mimics than NC group after osteogenic induction for 7 days (Fig. 5j). Collectively, the above findings proved that miRNA-141 was a negative regulator of SCAPs during the osteo/odontogenic differentiation.

### MiR-141 down-regulates SPAG9 expression in SCAPs

**Fig. 6:**
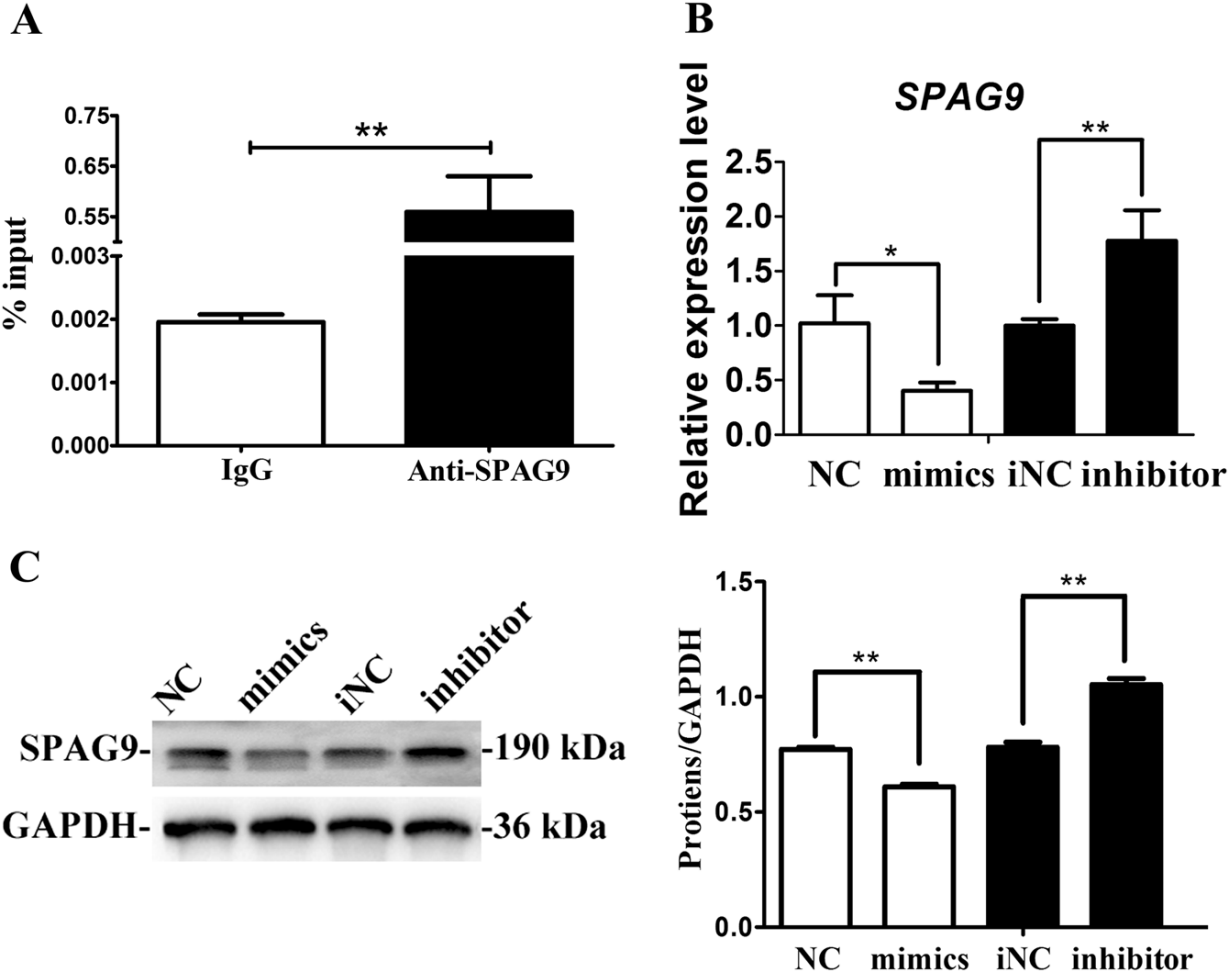
MiR-141 downregulates the expression of SPAG9. **a** Relative expression level of miR-141 in SCAPs was accessed by RIP assay (**P < 0.01). **b** Relative mRNA expression of SPAG9 in SCAPs transfected with with miR-141 mimics or miR-141 inhibitor (*P < 0.05, **P < 0.01). **c** Western blot analysis of protein expression of SPAG9 in SCAPs transfected with miR-141 mimics or inhibitor. **d** Results of western blotting was analyzed with ImageJ software and data were presented as ratio of target protein to GAPDH (**P < 0.01)

Previous study has revealed that miR-141 could markedly inhibit SPAG9 expression^23^. Furthermore, SPAG9 (also called C-jun-amino-terminal kinase-interacting protein 4, JIP4) is a scaffold protein that is important in the activation of p38 and JNK pathways, which are closely related to the osteo/odontogenic differentiation of SCAPs^15,27^. RIP assay demonstrated higher miR-141 expression n the anti-SPAG9 group compared with the anti-normal IgG group (Fig. 6a). As shown in Fig. 5b, qRT-PCR results revealed that *SPAG*9 level significantly decreased by miR-141 mimics and increased by miR-141 inhibitor (*P* < 0.05 or *P* < 0.01). Western blot also proved that miR-141 suppressed protein expression of SPAG9 in SCAPs (*P* < 0.01, Fig. 6c). The above findings confirmed SPAG9 is a direct target of miR-141.

### SPAG9 activates p38 and JNK pathways in SCAPs

**Fig. 7:**
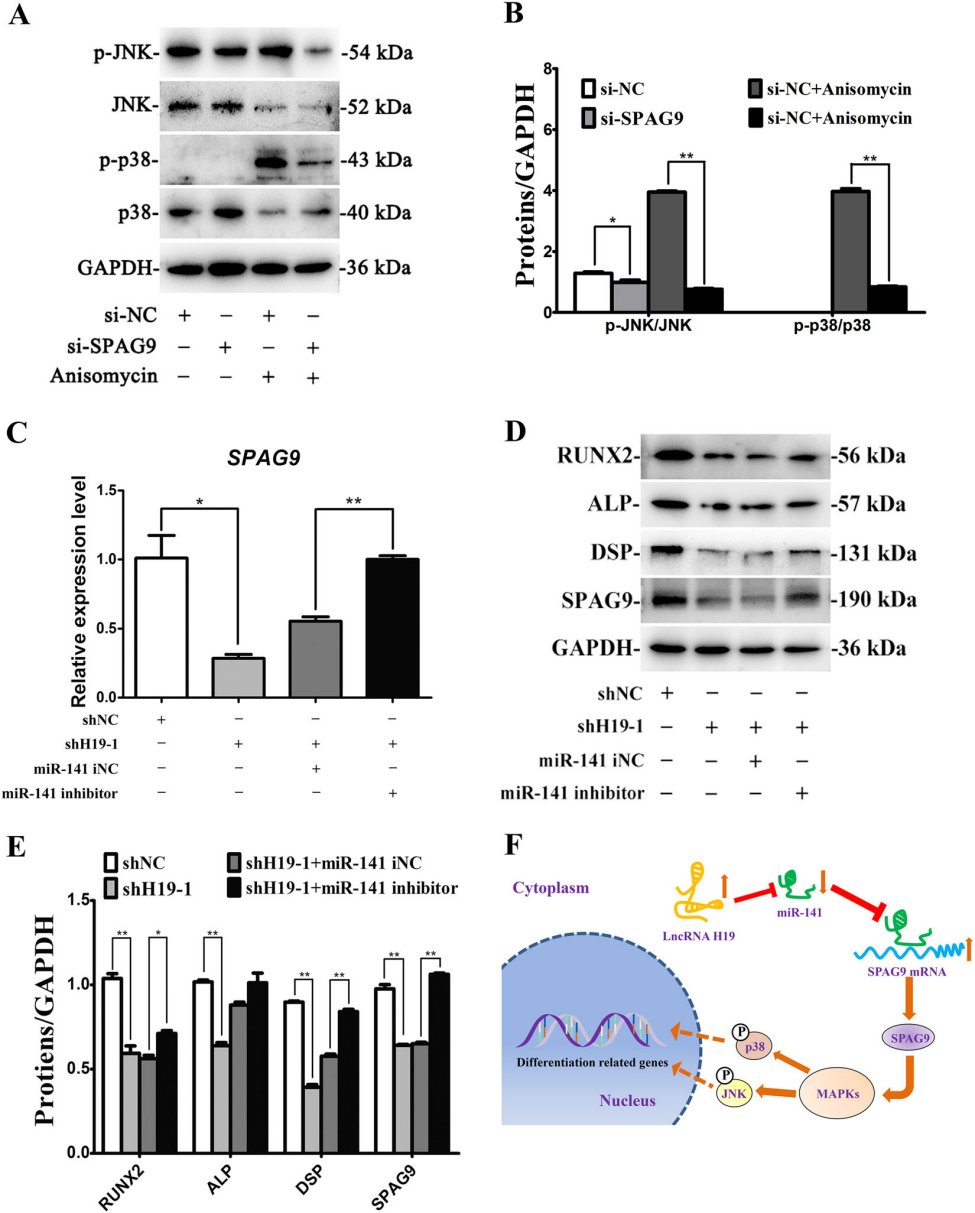
SPAG9 regulates p38 and JNK signaling pathways in SCAPs and the miR-141 inhibitor could rescue the shH19-1 mediated inhibitory effects of osteo/odontogenic differentiation in SCAPs. **a** Western blot assay for the expressions of genes relative to p38 and JNK signaling pathways at 72 h. **b** The ratio changes of p-p38/p38 and p-JNK/JNK at 72 h in different groups. Values were described as the means ± SD, n = 3. *P < 0.05, **P < 0.01. **c** Results of qRT-PCR analysis revealed the miR-141 inhibitor rescued the shH19-1 mediated downregulation of SPAG9 expression (*P < 0.05, **P < 0.01). **d** Results of western blot analysis indicated that the miR-141 inhibitor rescued the shH19-1 mediated downregulation of RUNX2, ALP, DSP, SPAG9. **e** Results of western blotting was analyzed with ImageJ software and data were presented as ratio of target protein to GAPDH in the form of grayscale value. (*P < 0.05, **P < 0.01). **f** Schematic diagram for lncRNA-H19/miR-141/SPAG9*/*MAPK axis

To determine whether SPAG9 could activate p38 and JNK pathways which exert a crucial role in the committed differentiation of SCAPs, relative proteins in SCAPs treated with si-SPAG9 at 72 h were investigated. Phosphorylated level of p38 in cytoplasm did not change in si-SPAG9 group as compared with si-NC group (Fig. 7a, b). However, the protein expression of phosphorylated JNK was obviously down-regulated in si-SPAG9 group compared with that in si-NC group. Co-treatment of the specific MAPK activator (anisomycin) reduced p-JNK/JNK in si-SPAG9 + anisomycin group compared with anisomycin treated SCAPs. Moreover, p-p38/p38 also remarkably decreased in si-SPAG9 + anisomycin group compared with anisomycin group (Fig. 7a, b). The above findings indicated that SPAG9 could activate p38 and JNK pathways.

### MiR-141 inhibitor can rescue the downregulated effects caused by H19 deficiency

To further investigate how miR-141 and SPAG9 were involved in H19-mediated differentiation, the rescure assays were performed. QRT-PCR results suggested that co-transfection with miR-141 inhibitor and shH19-1 significantly rescued *SPAG*9 expression in comparison to shH19-1 group (Fig. 7c). As presented in Fig. 7d, H19 deficiency-induced suppressed SPAG9, RUNX2, ALP, and DSP levels could be rescued in co-transfected cells (*P* < 0.05 or *P* < 0.01, Fig. 7e). In a word, lncRNA-H19 absorbs miR-141 as a ceRNA, increases the transcription of SPAG9, and activates the MAPK pathway to promote the committed differentiation of SCAPs (Fig. 7f).

## Discussion

In recent years, lncRNAs have received widespread attention as emerging regulators with diverse biological functions. LncRNAs are transcripts with over 200 nucleotides and take part in various cellular performances including cell growth, pluripotency and differentiation^28^. LncRNA-H19 locates near the telomeric region of chromosome 11p15.5 and is maternally imprinted^29^. It is abundantly expressed during fetal life, and is abundantly expressed and conserved non-coding transcripts in mammalian development. LncRNA-H19 participates in the osteogenic differentiation of BMSCs^26^. However, the underlying mechanism in osteogenesis regulation has not been completely studied.

We demonstrated how H19 served as an osteo/odontogesis-related lncRNA in SCAPs. H19 expression was upregulated during the osteogenic differentiation of SCAPs, indicating that H19 may provoke the osteogenic differentiation of SCAPs. Some researchers have shown that H19 has a positive influence on the proliferation of MSCs^30^. We did not observe the regulatory effect of H19 on SCAPs proliferation between H19 group and control group, which may be explained by the different cell lineages. Moreover, previous studies reported that H19 can plays an inhibitive role in cell proliferation, suggesting the effect of lncRNA-H19 on regulation of cell proliferation is complicated^31,32^.

Rescue experiments were conducted to explore the regulatory effect of lncRNA-H19 on the osteogenic differentiation of SCAPs. We found the osteo/odontogenic markers (e.g., ALP, RUNX2) were significantly upregulated in H19 overexpression group, which were downregulated after H19 knockdown. RUNX2 is part of the RUNX gene family, which has a primary function in osteoblast differentiation and directly stimulates the transcription of osteoblast-related gene (e.g., OSX and ALP)^33^. OCN is primarily generated by osteoblasts in the late stage of osteoblastic differentiation, and therefore its serum concentration could indicate bone formation^34^. As a specific marker of odontoblast, *DSPP* is mostly present in the secretory odontoblasts and DSP shows great influences in odontoblast differentiation and dentin mineralization^35^. In addition, ALP staining, alizarin red staining, and immunofluorescence staining assays further confirmed that H19 strongly promotes the osteo/odontogenic differentiation of SCAPs. Moreover, the in vivo study demonstrated that H19-overexpressing SCAPs displayed enhanced bone formation capacity.

Although H19 was demonstrated to be involved in the osteo/odontogenesis of SCAPs, the exact molecular mechanism remains to be elucidated. Recently, the “ceRNA” hypothesis is commonly accepted that lncRNA acquires functionality by acting as sponge of microRNA and abolishes microRNA’s inhibitory action to target mRNAs if there is a binding site between lncRNA and microRNA^21^. For instance, recent findings have demonstrated that H19 absorbs and antagonizes microRNAs in the *let-7* family, resulting in the downregulated protein-coding genes targeted by let-7^36^. In addition, H19 regulates mechanical tension-induced osteogenesis of BMSCs by absorbing miR-138 and up-regulates its downstream FAK^37^. LncRNA-H19 can enhance MSCs survival and angiogenic capacity by acting as a molecular sponge for miR-199a, eventually regulating in vitro expression of VEGFA^30^. To explore the underlying pro-oncogenic mechanism of H19 in this study, bioinformatics analysis indicated that miR-141 can bind to lncRNA-H19. MiR-141 belongs to the same cluster as the miR-200 family and has been observed to negatively regulate cellular senescence by inhibiting ZMPSTE24 expression^38^. However, the functions and potential targets of miR-141 in SCAPs remain to be fully elucidated. Thus, the effect of miR-141 on osteo/odontogenic differentiation of SCAPs and the relation between miR-141 and lncRNA-H19 were further explored. Firstly, our results verified that miR-141 negatively regulates osteo/odontogenic differentiation of SCAPs: miR-141 overexpression down-regulated mineral-related proteins/genes (OCN/*OCN*, OSX/*OSX*, RUNX2/*RUNX2*, ALP/*ALP*, DSP/*DSPP*) and matrix mineralization, whereas the miR-141 inhibitor up-regulated all of the mineral-related proteins/genes and matrix mineralization. Secondly, miR-141 expression was negatively regulated by H19: overexpression of H19 decreased miR-141 while H19 inhibition elevated miR-141 expression. To ascertain if there is direct binding between lncRNA-H19 and miR-141, luciferase reporter gene assay was conducted. MiR-141 inhibited the luciferase activity of vector containing H19 sequence. Mutation of the putative miR-141 target sites reversed the previous suppressive effect, indicating that lncRNA-H19 directly binds to miR-141 via the putative MRE in this “ceRNA” regulatory network.

MAPK signaling pathway exerts a vital role in mammals’ cellular regulations including apoptosis, proliferation, and differentiation^39^. C-Jun N-terminal kinase (JNK), extracellular signal-regulated kinase (ERK) and p38 MAPK are the major components of MAPK pathway^40^. Indeed, our previous studies have identified that MAPK pathway participates in osteo/odontogenic differentiation of tooth-derived MSCs^41–44^. It is reported that the 3′UTR of SPAG9 gene contains a sequence complementary to the miR-141 seed region, revealing miR-141 can directly target SPAG9^23^. Interestingly, the protein encoded by SPAG9 gene, also known as JIP4, is an important scaffold protein of MAPK pathway^27^. It activates MAPK pathway by regulating p38 and JNK phosphorlyation^45,46^. Hence, we hypothesized that lncRNA-H19 up-regulates SPAG9 by sponging miR-141, thus mediating osteo/odontogenic differentiation of SCAPs through MAPK pathway. To validate our hypothesis, RIP assays was performed at first. Results revealed that miR-141 could epigenetically inhibited the expressions of SPAG9 (Fig. 6a). Moreover, SCAPs transfected with miR-141 mimics downregulated the SPAG9 expression while miR-141 inhibitor transfection upregulated its expression (Fig. 6b, c). Collectively, all these data indicated that miR-141 targets the 3′UTR of the SPAG9 gene, suppressing mRNA translation of SPAG9 at the post-transcriptional level. Secondly, the interaction between MAPK pathway and SPAG9 was evaluated. As we expected, the protein expression of p-JNK was reduced by SPAG9 knockdown. Co-treatment with the specific MAPK activator (anisomycin) downregulated p-JNK/JNK and p-p-38/p-38 in si-SPAG9 + anisomycin group compared with anisomycin treated SCAPs, indicating that SPAG9 could activate the p-38 and JNK signaling pathways in SCAPs. To further validate whether lncRNA-H19 regulated SPAG9 expression by sponging miR-141 in osteo/odontogenic differentiation of SCAPs, we performed the rescue assays. Co-transfection of miR-141 inhibitor with shH19-1 significantly rescued *SPAG9* mRNA compared with the sh-H19 groups (Fig. 7c). Besides, the downregulation of miR-141 reversed the inhibitory effects of shH19-1 on the protein expressions of SPAG9 and other genes relative to osteo/odontogenic differentiation (Fig. 7d, e). Together, these results indicated that lncRNA-H19 epigenetically promoted SPAG9 transcription by interacting with miR-141.

To sum up, this study elucidated the osteogenic function of lncRNA-H19 in SCAPs, in which “lncRNA-H19/miR-141/SPAG9/MAPK” positive feedback loop plays a paramount role. Our results may provide references for revealing molecular mechanism of the odonto/osteogenic differentiation of SCAPs, and therapeutic targets in the future. However, whether other feedback loops take part in this ceRNA regulation are still needed to be further elucidated.

## Supplementary information

<b>Supplementary Figure.</b>

Supplementary Figure legends
