# Supplementary figures and images for: LncRNA H19 promotes the committed differentiation of stem cells from apical papilla via miR-141/SPAG9 pathway
Source: Cell Death Dis. 2019 Feb 12;10(2):130. doi: 10.1038/s41419-019-1337-3 (PMC6372621; doi:10.1038/s41419-019-1337-3)

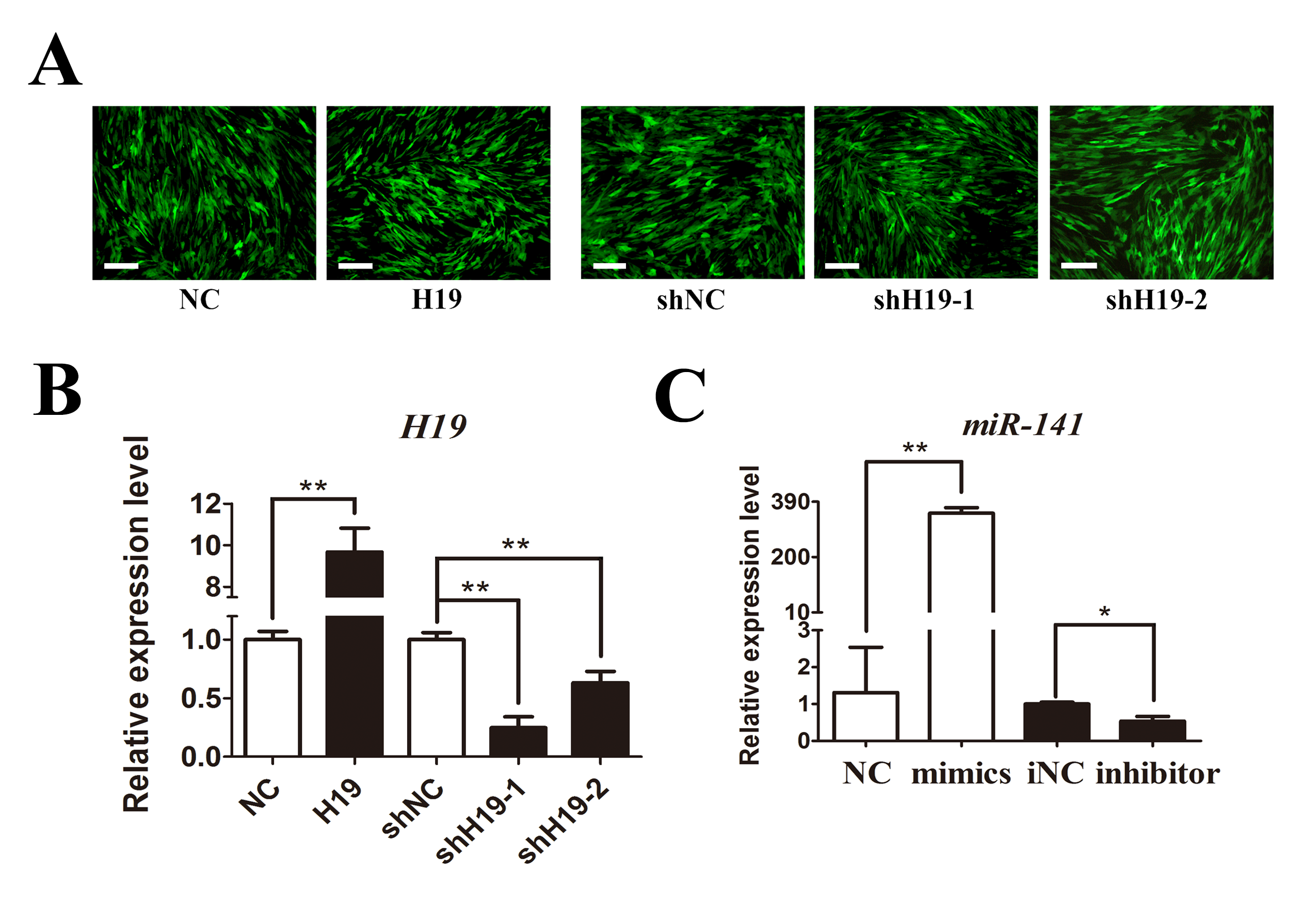

Supplement: Supplementary file 1 — Supplementary Figure. [file 41419_2019_1337_MOESM1_ESM.tif]
