# Supplementary material for: LncRNA H19 promotes the committed differentiation of stem cells from apical papilla via miR-141/SPAG9 pathway
Source: Cell Death Dis. 2019 Feb 12;10(2):130. doi: 10.1038/s41419-019-1337-3 (PMC6372621; doi:10.1038/s41419-019-1337-3)
Supplement: Supplementary file 2 — Supplementary Figure legends [file 41419_2019_1337_MOESM2_ESM.docx]

**Figure Legends**

**Supplementary Figure.** **Establishment of stably expressing transfectants by transfecting lentivirus, microRNA mimics and inhibitors.**

**A** Fluorescent photomicrographs showed lentivirus transduction in NC, H19, shNC, shH19-1 and shH19-2 groups. (Scale bar=50 μm). **B** Relative H19 expression measured by qRT-PCR in the H19 and shH19 groups. GAPDH was used for normalization. (***P*<0.01). **C** Relative miR-141 expression measured by qRT-PCR. U6 was used for normalization. Data were shown as the mean±SD (**P*<0.05, ***P*<0.01).
